# PW03-031 – Activation-induced cell death of human monocytes

**DOI:** 10.1186/1546-0096-11-S1-A257

**Published:** 2013-11-08

**Authors:** M Miranda-Garcia, J Daebritz, G Varga, T Weinhage, J Ehrchen, K Barczyk, J Roth, D Foell

**Affiliations:** 1 Pediatric Rheumatology and Immunology, Germany; 2 Dermatology, University Hospital Muenster, Muenster, Germany; 3 Institute of Immunology, University Hospital Muenster, Muenster, Germany

## Introduction

Monocytes are circulating cells with high plasticity. They respond to various stimuli with distinct activation and differentiation patterns, are able to secrete several humoral factors and they contribute to inflammation in the immune system, either by governing host defense response to invading pathogens or driving reactions to self-molecules in conditions of tissue-damage. Control of these mechanisms is necessary to ensure the self-limitation of inflammatory reactions and avoid perpetuated autoinflammation or autoimmunity. This aspect of immunoregulation is crucial and has been mainly associated with adaptive immunity. To date it is unclear how activated monocytes can regulate early cytokine signals promoting their survival or cell death.

## Objectives

The goal of the study was to explore the role of IL-1b and TNFa in activation-induced cell death (AICD) in human monocytes.

## Methods

Primary human monocytes were isolated and subjected to stimulation with GM-CSF and IFNg. Cell death was measured using Annexin V and propidium-iodide staining and analyzed by FACS. To explore the mechanism behind AICD of monocytes signaling pathways were analyzed by Western blot using the respective antibodies against phosphorylated and non-phosphorylated proteins. TNF-blockers were used to analyze the role of TNF in the process of AICD.

## Results

In the present study we demonstrate in vitro, that simultaneous treatment with GM-CSF and IFNy promotes AICD of human monocytes. Analyzing the signaling pathways that lead to cell death revealed that pyronecrosis is induced by GM-CSF and IFNg. Pyronecrosis has morphological characteristics of necrosis, is caspase- and RIP kinase1-independent but cathepsin-B-dependent. GM-CSF/IFNy-induced cell death of monocytes involved IL-1ß and TNFa-hypersecretion. Furthermore, pyronecrosis was found to be dependent on TNFa and could specifically be inhibited by TNF-blockers such as etanercept.

## Conclusion

Taken together, we identified AICD of monocytes as a novel mechanism, which could regulate inflammatory processes that may be altered in the context of autoinflammation. The involvement of different mediators and pathways in this process could have consequences on therapeutic strategies, e.g. for combination therapies involving TNF-blockers.

## Disclosure of interest

None declared

